# Ecological legacies of past human activities in Amazonian forests

**DOI:** 10.1111/nph.16888

**Published:** 2020-09-23

**Authors:** Crystal N.H. McMichael

**Affiliations:** ^1^ Department of Ecosystem and Landscape Dynamics Institute for Biodiversity and Ecosystem Dynamics University of Amsterdam 904 Science Park Amsterdam 1098 XH the Netherlands

**Keywords:** biodiversity, biomass, carbon storage, disturbance, forest dynamics, species enrichment or depletion, succession

## Abstract

In Amazonia, human activities that occurred hundreds of years ago in the pre‐European era can leave long‐lasting effects on the forests – termed ecological legacies. These legacies include the intentional or nonintentional enrichment or depletion of certain species. The persistence of these legacies through time varies by species, and creates complex long‐term trajectories of post‐disturbance succession that affect ecosystem processes for hundreds of years. Most of our knowledge of Amazonian biodiversity and carbon storage comes from a series of several hundred forest plots, and we only know the disturbance history of four of them. More empirical data are needed to determine the degree to which past human activities and their ecological legacies affect our current understanding of Amazonian forest ecology.

1

**Table:** 

**Contents**		
	Summary	1
I.	Introduction	1
II.	Ecological legacies on forest composition	2
III.	Ecological legacies on biomass and carbon dynamics	3
IV.	Outlook: advancing our knowledge of long‐term ecological legacies	3
	Acknowledgements	4
	References	4

## 2 Introduction

The importance of Amazonian rainforests for an array of ecosystem services and functions is well known amongst scientists but perhaps less so amongst policymakers (Levis *et al*., 2020). The biodiversity of Amazonian forests is immense (ter Steege *et al*., 2020), but the mechanisms driving the relative abundances and distributions of this diversity remain largely unresolved. Environmental gradients, biotic interactions and dispersal limitation all play a role in structuring diversity patterns in Amazonian forests (e.g. Wright, 2005). An emerging hypothesis is that past disturbances in the landscape, particularly those caused by human activities, have also played a role in shaping the structure, function and diversity patterns observed in modern forests (Levis *et al*., 2017; McMichael *et al*., 2017b).

People have lived in Amazonia for over 10 000 yr (Roosevelt, 2013) and have cultivated maize in some regions for over 6000 yr (Brugger *et al*., 2016; Bush *et al*., 2016). Besides cultivation, people in the pre‐European era also used fire to clear forests and amend soils, and they domesticated several plant species (e.g. Neves & Petersen, 2006; Piperno, 2011; Clement *et al*., 2015). Some of these forests have been managed continually by indigenous people for hundreds or even thousands of years, sometimes termed intensive or opportunistic agroforestry (Neves, 2013; Levis *et al*., 2018). But many areas that were cleared and managed at the time of European arrival *c*. 500 years ago were abandoned, when a majority of indigenous populations collapsed (Denevan, 2014). Following European colonization, many Jesuit missions were established but were quickly abandoned (Reeve, 1993). The ‘Amazonian rubber boom’ (*c*. ad 1850–1920) was a subsequent influx of European colonists that later collapsed because establishing rubber plantations was cheaper in Malaysia (Hecht, 2013). It is likely that all of these past waves of colonization and abandonment in the landscape have left ecological legacies on the forests, where trees often have life spans exceeding 150 yr.

Ecological legacy refers to the influence of an event (i.e. disturbance) on an ecosystem and its persistence over a given time period, and is a term that has been widely used in succession studies. The type and intensity of human disturbance (e.g. clear cut versus forest burning) affect the trajectory of the ecological legacy in Amazonian systems on decadal timescales (e.g. Mesquita *et al*., 2015). The long‐term ecological legacies of past human impacts during the pre‐ and post‐European eras, however, remain more obscure. Here I review recent advances in our understanding of long‐term ecological legacies in Amazonia with a focus on biodiversity and carbon storage, and highlight why assessing past disturbances is crucial for understanding the patterns and dynamics observed in these globally important forests.

## 3 Ecological legacies on forest composition

**Fig. 1:**
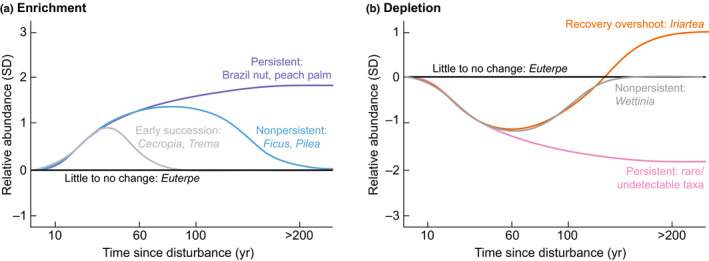
The long‐term effects of human disturbances on Amazonian tree species. (a) The enrichment of taxa can be either intentional or nonintentional, and can be persistent or nonpersistent through time. (b) The depletion of taxa can also be persistent or nonpersistent, although sometimes the recovery of these taxa overshoots the abundances before the disturbance. Some taxa, while useful, show no real change in abundances with low levels of past human disturbance.

Most studies of ecological legacies on Amazonian forest composition have focused on the enrichment and long‐term persistence of useful species. It has been suggested that *Bertholettia excelsa* (Brazil Nut), *Bactris gasipaes* (Peach Palm) and other edible plants were enriched in the pre‐European era, and their abundances have remained artificially high ever since (i.e. for hundreds of years) (Fig. 1a; Scoles & Gribel, 2011; Clement *et al*., 2015; Thomas *et al*., 2015; Maezumi *et al*., 2018). In a series of *c*. 1100 forest plots in Amazonia, there were higher richnesses and abundances of domesticated tree species in locations that were closest to known pre‐European archaeological sites (Levis *et al*., 2017). Many of these same domesticated species that show a relationship with pre‐European occupation are also some of the most abundant across the basin (ter Steege *et al*., 2013).

Ecological legacies following disturbances may not always be persistent, as is the case with early successional taxa, such as *Cecropia* or *Trema* (Fig. 1a). Mid‐ to late‐successional taxa, such as *Ficus* and *Pilea*, have longer life spans and can persist for centuries, but eventually decrease in abundance (Åkesson *et al*., 2020). In Costa Rican forests, the proportion of old‐growth taxa can reach 30–40% within 25–30 ys following a disturbance, but then only reaches 50% at 80 yr following a disturbance (Chazdon *et al*., 2009). The systems are expected to continue shifting in their composition for at least 200 yr following a disturbance (Foster, 1990; Loughlin *et al*., 2018). These nonpersistent ecological legacies are often simply part of the long‐term successional process.

Ecological legacies in Amazonia can also include the depletion of species by people (Fig. 1b). The most commonly observed example of species depletion in palaeoecological records is the palm *Iriartea deltoidea*, which occurs in higher abundances where there is little to no evidence of human activity compared with areas containing past fire and cultivation (Bush & McMichael, 2016; Heijink *et al*., 2020). *Iriartea deltoidea* usually recovers *c*. 100 yr after site abandonment and often reaches abundances higher than before the disturbance (Fig. 1b). *Iriartea deltoidea* is currently the sixth most common tree species in Amazonia (ter Steege *et al*., 2020), and it is possible that this rise to dominance occurred as result of recovery from past depletions. It is hard to find examples of persistent depletion, which would require a species to have poor recruitment and limited seed dispersal. These types of species are rare in the landscape (Wills *et al*., 1997), and therefore almost undetectable using palaeoecological reconstructions.

Palms are disproportionately abundant in Amazonia compared with other tree families, and have varying degrees of responses to human disturbances. *Wettinia* is a genus of mid‐successional palms that has a similar, nonpersistent, negative response to human disturbance like *I. deltoidea*. *Wettinia*, however, does not seem to have the recovery overshoot that has been documented in *Iriartea* (Fig. 1b; Åkesson *et al*., 2020). The palm genus *Euterpe* includes the first and seventh most common tree species in Amazonia (ter Steege *et al*., 2013). Both of these *Euterpe* species are useful for their fruit, but their abundances do not seem to shift drastically in response to low levels of human disturbance (Fig. 1; Heijink *et al*., 2020).

## 4 Ecological legacies on biomass and carbon dynamics

Amazonia provides a significant input to global carbon and climate models, and is believed to sequester more carbon than it releases (i.e. is a carbon sink; e.g. Aragao *et al*., 2014). Global climate and carbon models assume that forests are not recovering from past disturbances, although this is intensely debated (Wright, 2013). Over recent decades, the carbon sequestration potential of Amazonia has been declining because increases in tree productivity rates have slowed and mortality rates have increased (Brienen *et al*., 2015). The effects of short‐term disturbances (e.g. El Niño events) have been studied (Phillips *et al*., 2009), but very little is known about the longer‐term disturbance histories within the forest plots that are used to estimate Amazonian carbon dynamics.

**Fig. 2:**
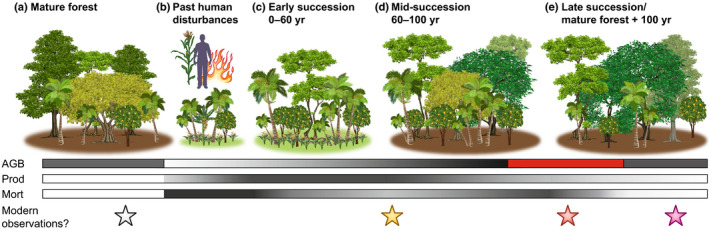
It is unknown where modern observations lie in the context of long‐term successional trajectories. (a) Mature forests have more large trees, fewer understorey trees and few grasses (brown forest floor). (b) Past human disturbances include fire, forest clearance, cultivation, and tree domestication (increased palms and fruit trees). Canopy openings result in a thicker understorey, increased numbers of grasses (green forest floor) and pioneer taxa. (c) Early successional forests retain high numbers of domesticated species, palms and pioneers, and begin accumulating large trees. (d) Mid‐successional forests retain high abundances of domesticates, long‐lived pioneers and large trees, resulting in higher biomass than mature forests (red bar, above‐ground biomass (AGB)). (e) Pioneers die off and mature forests re‐emerge, although they are compositionally different than before the disturbance. Darker shading indicates higher values and lighter shading indicates lower values for changes in AGB, productivity (Prod), and mortality (Mort) through time following a large‐scale disturbance.

Old growth forests typically contain high amounts of biomass, but have relatively low productivity and mortality rates (Fig. 2a). Landscape modifications by people lower the biomass but increase the productivity and mortality of the system until the disturbance ceases (Fig. 2b). Of these modifications, fire and deforestation are the most intense, and biomass recovery patterns are known to be linked to disturbance intensity (de Avila *et al*., 2018). Early successional species transition to mid‐successional species, which have a higher biomass, *c*. 60 yr after abandonment, and this process can happen for over 100 yr (Fig. 2c; Loughlin *et al*., 2018). Biomass recovery, however, has been shown to exceed 100% of the pre‐disturbance values until at least 100 yr following an event (Fig. 2d; Poorter *et al*., 2016). There are no current estimates of how long it takes for the long‐lived, mid‐successional species to die off and for biomass to return to pre‐disturbance values (Fig. 2e). There are also no data yet as to how long‐term succession may be affecting the forest dynamics observed in recent decades.

It is possible that the decline of the Amazonian carbon sink and slowing down of productivity observed in the last 30 yr (Brienen *et al*., 2015) reflect biomass and carbon dynamics returning to pre‐disturbance values over the last several hundred years (Fig. 2d,e). Biomass and carbon dynamics are directly linked with species composition (e.g. Phillips *et al*., 2019), and thus ecological legacies of species composition (Fig. 1) probably translate to legacies on biomass and carbon dynamics (Fig. 2). High abundances of *Bertholettia excelsa* in southwestern Amazonia, which may be related to past human enrichment (Fig. 1a), play a large role in the overall carbon storage potential of those forests (Selaya *et al*., 2017). The large changes in palm abundances seen over the last several thousand years (Bush & McMichael, 2016) have also probably affected biomass and carbon dynamics. The forest plots used to measure carbon dynamics in Amazonia are disproportionately located in areas containing high densities of archaeological sites and high probabilities of pre‐European settlement (McMichael *et al*., 2017b). These plots are thus probably capturing changes in carbon dynamics related to long‐term successional dynamics and ecological legacies.

## 5 Outlook: advancing our knowledge of long‐term ecological legacies

There are several knowledge gaps and debatable aspects regarding ecological legacies in Amazonian forests. The first concerns the timing and intensity of the disturbance that created the ecological legacy. Most research has focused on linking pre‐European human activities with modern vegetation, but the impacts of the last 400 yr of postcolonial activities are also beginning to be considered (McMichael *et al*., 2017a; Arienzo *et al*., 2019). These two eras had different types and intensities of land use, which affect long‐term successional trajectories (Bodin *et al*., 2020).

**Fig. 3:**
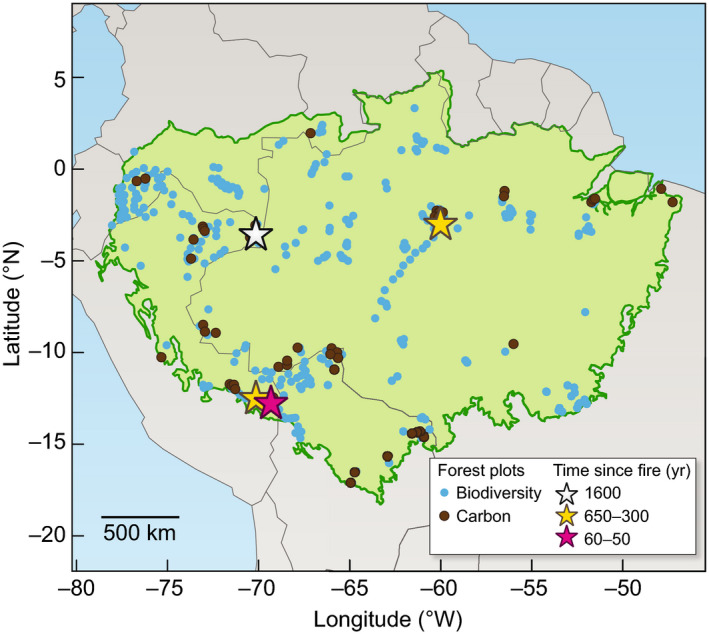
Map showing the distribution of Amazonian forest plots that are used to observe biodiversity (blue circles) and carbon dynamics (brown circles). Stars represent forest plots where there is information on the time since the last fire (see Fig. 2).

The time since the last major disturbance is almost unknown in the forest plots used to study biodiversity and carbon dynamics. The time since the last fire has been published in only four out of the hundreds of surveyed forest plots (Fig. 3; Heijink *et al*., 2020). Los Amigos in Peru has burned in some areas as recently as 50 yr ago (Figs 2, 3, yellow star), whereas Amacayacu in Colombia has not burned in over 1600 yr (Figs 2, 3, white star). The other two forest plots had burned between 300 and 600 yr ago, and it is unknown whether biomass and composition have returned to pre‐disturbance values (Figs 2, 3, pink and red stars). Interestingly, palm abundances in the modern vegetation and in vegetation reconstructions were significantly lower at Los Amigos, which has had more recent and frequent fire events over the last 4000 yr compared with the other plots (Heijink *et al*., 2020). The timing of the last major disturbance for the majority of these forest plots remains unknown (Fig. 3).

The spatial extent of these past human activities and ecological legacies into less well‐studied and less accessible regions of the forest also remains unknown and is highly debated. Some have argued that the extent of site abandonment and subsequent forest regrowth after European arrival was so great that it caused a global decrease in CO_2_ concentrations (Koch *et al*., 2019). But these assumptions rely on archaeological datasets, which, like the forest plots, are biased towards the accessible areas in Amazonia (McMichael *et al*., 2017a). Many soil surveys conducted in randomized and less accessible areas show little to no evidence of past fire or human occupation, or even the slightest bit of past forest opening (Piperno *et al*., 2019). Despite extensive scanning of hundreds of samples for charcoal in soils collected from a forest plot in the Colombian Amazon, only three were collected that were > 10 mg, or the minimum size required for ^14^C dating (Heijink *et al*., 2020). There was no evidence of maize or past forest openings in the 90 phytolith samples analysed from this forest plot, and the most recent fire occurred 1600 yr ago (Figs 2, 3; Heijink *et al*., 2020). The probability of the modern vegetation reflecting past human activities, or an ecological legacy, at this site is almost zero.

The integration of ecological, palaeoecological, and archaeological data are crucial to understanding the long‐term ecology and ecological legacies in Amazonian forests. Archaeologists and palaeoecologists are beginning to collect complementary datasets (Mayle & Iriarte, 2014; Maezumi *et al*., 2018; Åkesson *et al*., 2019). But to fully understand how past human activities affect modern processes, the palaeoecological and archaeological data must also be collected within the series of ecological surveys – the Amazonian forest plots that are used for estimating biodiversity and carbon dynamics. The four plots with past fire and vegetation data tell radically different stories, and filling in the gaps on the continuum of past disturbances is necessary to make links with the patterns found in the modern observational data (Figs 1, 2, 3).

Advancements in techniques of looking into the past are pushing the boundaries of what can be learned from ecological, palaeoecological and archaeological datasets. One example is by extracting dendrochronological, isotopic and genetic information from living trees, and using that information as time capsules of past human and climatic change (Caetano‐Andrade *et al*., 2020). Another example is by using the chemical and morphological composition of charcoal found within palaeoecological and archaeological archives to infer the temperature (intensity) of past fires and the types of plant material that were burned (Goulart *et al*., 2017; Gosling *et al*., 2019). These technical developments, as well as those geared towards improving the taxonomic identification of macro‐ and microfossils, are providing deeper insights into how past disturbances are manifested in modern systems.
